# A Decontamination Process to Remove Metals and Stabilise Montreal Sewage Sludge

**DOI:** 10.1100/tsw.2002.201

**Published:** 2002-04-26

**Authors:** G. Mercier, J.F. Blais, F. Hammy, M. Lounes, J.L. Sasseville

**Affiliations:** Institut National de la Recherche Scientifique (INRS-Eau), Université du Québec, 2700, rue Einstein, Sainte-Foy, Quebec, Canada; Biolix Corporation, Iberville II, 1175 Lavigerie, Bureau 50, Sainte-Foy, Quebec, Canada G1V 4P1,

**Keywords:** biosolids, leaching, metal, odour, sewage sludge, stabilisation

## Abstract

The Montreal Urban Community (MUC) treatment plant produces approximately 270 tons of dry sludge daily (tds/day) during physicochemical wastewater treatment. The sludges are burned and contribute to the greenhouse effect by producing atmospheric CO_2_. Moreover, the sludge emanates a nauseating odour during its thermal stabilisation and retains unpleasant odours for the part (25%) that is dried and granulated. To solve this particular problem, the treatment plant authorities are currently evaluating an acidic chemical leaching (sulfuric or hydrochloric acid) process at a pH between 2 and 3, using an oxidizing agent such as ferric chloride or hydrogen peroxide (METIX-AC technology, patent pending; [20]). They could integrate it to a 70 tds/day granulated sludge production process. Verification of the application of METIX-AC technology was carried out in a pilot plant set up near the sludge production plant of the MUC. The tests showed that METIX-AC technology can be advantageously integrated to the process used at the MUC. The residual copper (274 ± 58 mg/kg) and cadmium (5.6 ± 2.9 mg/kg) concentrations in the treated sludge meet legislation standards. The results have also shown that odours have been significantly eliminated for the dewatered, decontaminated, and stabilized biosolids (> 97%) compared to the non-decontaminated biosolids. A high rate of odour elimination also was obtained for the liquid leached biosolids (> 93%), compared to the untreated liquid biosolids. The fertilising value (N and P) is well preserved by the METIX-AC process. Dissolved organic carbon measurements have showed that little organic matter is brought in solution during the treatment. In fact, the average concentration of dissolved organic carbon measured in the treated liquid phase is 966 ± 352 mg/l, whereas it is 1190 ± 325 mg/l in untreated sludge. The treated sludge was first conditioned with an organic polymer and a coagulant aid. It was successfully dewatered with various dehydration equipments (filter press, rotary press, centrifuge).

### INTRODUCTION

Agricultural utilization of sewage sludges is wide[1]. However, it can contaminate soils, cause phytotoxicity, and pollute the environment[2,3]. Elevated concentrations of heavy metals exist in many agricultural soils following sewage sludge applications[4,5]. Standard technologies of sludge treatment, like aerobic or anaerobic digestion, do not remove metals. Two different methods, source control or removal at the treatment plant, can accomplish metal concentration reduction. Even if it is possible to reduce metal concentrations by source control, it is difficult and sometimes impossible to reach required levels when the norms are strict. Many studies have shown that a large proportion of metals come from domestic houses and street runoff[6,7].

#### METIX Technologies

A research team of INRS-Eau (Quebec, Canada) has developed various processes for metals removal from sewage sludge over the last decade. Two biological processes have been developed, one using elemental sulphur[8], and the other one using ferrous sulphate[9]. These processes remove, at low cost, a large proportion of metals, odours, and pathogenic organisms in the sludge without diminishing the fertilising value[9,10,11,12,13,14,15]. These processes have been tested all the way from the laboratory scale to the industrial pilot plant at the wastewater treatment plant of the Valcartier army base (Quebec)[16,17]. Neural network modelling on the METIX process using *Thiobacillus ferrooxidans* bacteria have also been made[18].

#### Montreal Urban Community (MUC) Treatment Plant

The MUC wastewater plant uses a physicochemical process; thus, it precipitates phosphorus and the settlable solids from the wastewater by the addition of ferric chloride or aluminium sulphate. The sludge is then sent to homogenisation tanks prior to being pumped to ten filter presses and five Fournier rotary presses for dewatering. This step brings the total solid content from around 3% (liquid) to around 33% (solid). The resulting solid is then burnt in four different incinerators. Recently, MUC wastewater plant bought granulation dryers (thermic stabilisation) to treat 70 tds/day. The dry pellets obtained could be sold on the market for farming, tree growing, or gardening. The market price is estimated to be around U.S.$44/tds, but if sold in 22 kg bags, the value of decontaminated sludge varies between U.S.$125 and 312/tds. However, metal concentrations for granulated sludges are over the permitted limits for use on farms, forests, and gardens[19]. This sludge also emits nauseating odours during the drying process, causing problems with the population living near the treatment plant. The MUC wastewater plant authorities solicited the Consortium Biolix-Mabarex and INRS-Eau to test the METIX-AC technology. Hence, the INRS-Eau pilot plant has been transported close to the MUC wastewater treatment plant. This paper shows briefly the results of this pilot plant test.

### MATERIALS AND METHODS

#### Pilot Plant and Process

The pilot plant is in a trailer of 3.7 × 18.5 m. Tests were carried out to show the efficiency of the process and to find the best design to treat the MUC sludge. METIX-AC is a chemical leaching process that is in the process of being patented[20]. It uses an acid (sulphuric or hydrochloric) to maintain pH between 2 and 3, and a strong oxidiser (ferric chloride, hydrogen peroxide, potassium permanganate, or ozone) to maintain an oxido-reduction potential (ORP) of at least +400 mV. METIX-AC was applied in an agitated reactor of 4 $m^3$. Control of the chemical quantities added to the sludge (acid and leaching agent) was made through the use of pH controller and an ORP electrode. Type and quantity of leaching agents, pH, and residence time in the reactor were the main parameters tested, but no more details can be given as the process is patent pending. After leaching, the treated sludge was conditioned with an organic polymer and filtered on a filter press (Thames Technologies model 800 mm, Grapevine, Texas, U.S.). The most appropriate polymer and its dosage were chosen according to the size and solidity of the floccules formed. When the appropriate polymer and dosage are optimal, one can dewater the sludge by pressing it in a cotton cloth between the hands. A capillary suction timer or viscosity meter can also be used, but they were not for this study.

The filter press had 12 chambers for a total filtering surface of 8.58 $m^2$. The filter press cloths had pores of 50 to100 µm, allowing flow of 0.85 standard $m^3$ of air per minute. The maximum operating pressure was 100 psi, and the 12 chambers contained 170 l of sludge. Dewatering assays also were done with a pilot plant centrifuge (of the company Pieralisi, subsidiary of Degremont (Ondeo), Montreal, Canada). The speed of the centrifuge was fixed at 3,500 rpm, and the applied flow rate was 2.25 $m^3$/h. A rotary press was rented (Fournier company, 1,200 mm, Black Lake, Québec, Canada) to dewater the decontaminated sludge. The flow rate varied between 3 and 4.2 $m^3$/h. The sludge cake was neutralized with hydrated lime before it was sent to a landfill. The liquid fraction (filtrate) was sent to a precipitation tank, where the addition of a lime solution increased the pH and caused precipitation of the metals. The metal hydroxide precipitate was decanted, and the precipitate was filtered (Thames Technologies filter press, model 470 mm (Thames Technologies, Grapevine, Texas, U.S.) four chambers, total filtering area of 1.18 $m^2$ and 5.6 l of cake capacity). The supernatant was sent to the sewer.

### Sampling and Analysis

Liquids samples were first filtered on Whatman 934-AH filter paper, and filtrates were acidified by adding 5 (v/v) of HCl. Samples of solids and liquids were stored at 4°C and analysed. The ORP (platinum electrode) and pH (glass electrode) were also monitored. Total solids (TS) and suspended solids (SS) were measured according to APHA protocol[21]. Solids and metallic residues were dissolved in a mixture of strong acids and peroxide ($HNO_3$, HCl, and $H_2O_2$ 30%, according to the protocol used by Quebec Ministry of Environment)[22]. Metal content (Al, Cr, Cu, Fe, Mn, Ni, Zn) and macronutrient content (P, Ca, Na, K) solubilised were analysed by inductively coupled plasma spectrophotometry (ICP-AES, Varian model Vista-AX, Missisauga, Ontario, Canada). Concentrations of Cd and Pb were measured by flame atomic absorption spectrophotometry (Varian model SpectrAA-20, Missisauga, Ontario, Canada). Nitrogen, carbon, and sulphur concentrations of the dewatered treated sludge were measured with a thermal analyser (Carlos Erba Instruments model NA1500, CE Instruments, Wigan, U.K.). Dissolved organic carbon (DOC) in liquids was analysed by a combustion analyser and detection performed by infrared detector (Model TOC-5000A, Shimadzu, Columbia, MD, U.S.), and ammonium was determined with a colorometric autoanalyzer (model AA2, Technicon Corporation, New York, U.S.). The company Odotech (Montréal, Canada) performed odour tests according to the ASTM E679-91 standard procedure[23].

## RESULTS AND DISCUSSION

Twenty-seven batch tests were performed on MUC sludge. The volume of each assay varied from 2 to 12 $m^3$. Overall, 132 $m^3$ of sludge or 3 tds were treated. Between 21.9 to 38.9 g/l (average of 29.5 ± 2.9 g/l) of total solid content were measured in the sludge. Initial pH range was 6.08 to 6.7. Initial sludge was anoxic with an ORP from –175 to –70 mV (average of –122 ± 25 mV). Appropriate quantities of acid and leaching agents must be added to obtain leaching conditions sufficient (pH 2 to 3 and ORP over 400 mV) to solubilise metals without solubilising N, P, and organic matter. The exact quantities are part of the patent on METIX-AC, which is pending[20]. The metal solubilisation was easily achieved during pilot plant testing and only minor engineering problems were enountered.

The first 13 tests were done using the filter press, and 12 others tests were accomplished using the Fournier rotary press. A centrifuge was used for the last two tests. Table 1 presents the results concerning metals removal. Cu and Cd are the main pollutants in this sludge as they exceeded the limits accepted for dried granulated sludge[19]. The concentrations of Cu and Cd were reduced to values below the objective of 430 mg Cu/kg and 10 mg Cd/kg. The decontaminated sludge contained an average of 5.61 ± 2.93 mg Cd/kg and 274 ± 58 mg Cu/kg, demonstrating that the process is working well.

### Odour Elimination

According to the threshold of odour detection with various dilution rates[23] (ASTM E679-91, ASTM 1999), the Odotech procedure technique used in this work consisted of measuring the flow of odour emitted per unit of surface area. A qualified jury (independent persons having an appropriate level of odour detection) of six persons was employed. Table 2 presents the results of these tests. High percentages of odour flux elimination were obtained, between 97.1% and 99.7% for treated dewatered sludge and 93.6% for liquid treated sludge. Values for treated dewatered sludges were compared to untreated dewatered sludges, and values for treated liquid sludge were compared to the values of untreated liquid sludge.

### Effect on the Fertilising Value and Solubilisation of Organic Matter

The METIX-AC process has little effect on the fertilising value. The dewatered treated sludge contained total nitrogen (TKN) of 2.94 ± 0.52% compared to 2.28 ± 0.79% in dewatered untreated sludge. Soluble ammonium concentration was 155 ± 77 mg/l for treated sludge compared to 265 ± 121 mg/l for untreated sludge. For total P, the same conclusion applies (1.64 ± 0.23% compared to 1.73 ± 0.28%). Soluble P increased from 1.38 ± 2.81 mg/l for untreated sludge to 17.5 ± 5.9 mg/l for treated sludge. Soluble K increased from 49.8 ± 14.2 mg/l to 85.9 ± 13.7 mg/l,

**TABLE 1**
**Metal Concentrations in Sludge Before and After Decontamination (Dewatered Sludge)**

| Metals | Initial concentration (mg/kg) | | Final concentration (mg/kg) | Objective* (mg/kg) |
|---|---|---|---|---|
| | Range | Mean | | |
| Al | 10,300–17,900 | 14,000 ± 2,000 | 8,300 ± 1,600 | ** |
| Cd | 7.2–15.7 | 10.8 ± 2.3 | 5.61 ± 2.93 | < 10 |
| Cr | 82.8–232 | 155 ± 39 | 129 ± 37 | < 1,060 |
| Cu | 317–568 | 472 ± 60 | 274 ± 58 | < 430 |
| Fe | 41,900–97,000 | 73,200 ± 17,900 | 68,600 ± 18,300 | ** |
| Ni | 26.6–77 | 44.4 ± 10.3 | 29.5 ± 9.5 | < 180 |
| Pb | 41.1–141 | 91.8 ± 18.9 | 99.0 ± 18.6 | < 500 |
| Zn | 433–817 | 608 ± 100 | 292 ± 120 | < 1,850 |

* Temporary limits as suggested by the Bureau de Normalisation du Québec
** [Al] + 0.5 [Fe] should be inferior to 100,000 mg/kg

**TABLE 2**
**Odour Elimination Measurements During the Sludge Decontamination**

| Tests | Concentration (u.o.*/m$^3$) Initial | Concentration (u.o.*/m$^3$) Final | Odour flux (u.o./(m$^2$.s)) Initial | Odour flux (u.o./(m$^2$.s)) Final | Odour flux elimination (%) |
|---|---|---|---|---|---|
| Dewatered sludge | | | | | |
| A | 197,393 | 2,960 | 170.95 | 2.56 | 98.5 |
| B | 195,569 | 5,663 | 169.38 | 4.9 | 97.1 |
| C | 237,865 | 5,146 | 206.01 | 4.46 | 97.9 |
| D | 200,749 | 621 | 173.86 | 0.54 | 99.7 |
| E | 42,915 | 1,244 | 37.17 | 1.08 | 97.1 |
| Non-dewatered sludge | | | | | |
| F | 189,518 | 12,130 | 164.13 | 10.51 | 93.6 |

* u.o.: unit of odour but this soluble element is also lost when dewatering untreated sludge. Total carbon of the sludge is not lost in the process (26.86 ± 7.79% vs. 33.41 ± 4.41%, w/w). The DOC decreased from 1190 ± 325 mg/l for untreated sludge to 966 ± 352 mg/l for treated sludge, thus showing no dissolution of organic matter by the low pH.

#### Sludge Dewatering

Dewatering is an important step for any treatment process, particularly when conditioning sludge at low pH. Much research has been required in the past to find an appropriate conditioning agent and dosage[17,24]. The filter press cake contained 22.7 ± 4.9% TS (w/w) with a high quality effluent (0.72 ± 0.23 g/l of SS) and the best retention of solids obtained (97.1 ± 1.6% SS). Rotary pressing produced a drier cake (37.3 ± 9.9% TS) but gave a filtrate with 1.66 ± 0.57 g/l of SS and retention of solids of 92.9 ± 2.2%. Centrifugation was also efficient with a cake containing 22.4 ± 2.6% TS. Solid retention with the centrifuge was 91.8 ± 1.4% with 2.22 ± 0.38 g/l of SS in filtrate.

### CONCLUSIONS

This pilot plant study shows that the METIX-AC process is efficient to remove metals, eliminate odours, and keep the fertilising value of the MUC wastewater sludge. Encountered engineering problems related to the implementation of this process were easily solved. Appropriate polymers have been found, and filter press, rotary press, or centrifuge can be used for dewatering. METIX technologies have been previously demonstrated for treating biological sludges, and following this project, the METIX-AC process can be installed to treat the physicochemical MUC sludge.

### ACKNOWLEDGEMENTS

We thank the Montreal Urban Community for providing financial support to carry out this work. Sincere thanks are also due to Jean-Philippe Raboud from Mabarex Inc. for his technical assistance.
